# Multimodal imaging features of retroperitoneal anastomosing hemangioma: a case report and literature review

**DOI:** 10.3389/fonc.2023.1269631

**Published:** 2023-10-25

**Authors:** Liqing Zhang, Jian Wu

**Affiliations:** ^1^ Department of Radiology, Affiliated Hangzhou First People’s Hospital, Zhejiang University School of Medicine, Hangzhou, China; ^2^ Department of Pathology, Affiliated Hangzhou First People’s Hospital, Zhejiang University School of Medicine, Hangzhou, China

**Keywords:** retroperitoneal, anastomosing hemangioma, vascular tumors, magnetic resonance imaging, computed tomography

## Abstract

**Background:**

Anastomotic hemangioma is a rare subtype of capillary hemangioma primarily found in the genitourinary tract. We present a case of a patient with an anastomotic hemangioma located in the retroperitoneal space; then, we explore and summarize the imaging features from previously reported cases for accurate diagnosis.

**Case presentation:**

A 57-year-old woman complained of left lower back pain. Contrast-enhanced ultrasound revealed a hypoechoic mass with “slow-in and slow-out” enhancement. Abdominal CT scan displayed a well-defined, round soft tissue mass in the right retroperitoneal region with obvious enhancement. MRI indicated low signal on T1-weighted imaging, high signal on T2-weighted imaging and diffusion-weighted imaging, and progressive enhancement after enhancement. Surgical removal of the tumor was performed. Histopathological examination exhibited a distinct tumor border with interconnected blood vessels and a cavity lined by a single layer of cubic endothelial cells. Immunohistochemistry confirmed the presence of CD31[+] and CD34[+]. The final pathological diagnosis was anastomotic hemangioma. No recurrence was observed during a 40-month follow-up.

**Conclusion:**

Retroperitoneal anastomotic hemangioma is a rare and benign neoplasm that may be misdiagnosed as ectopic pheochromocytoma or angiosarcoma. This case report presents and analyzes the imaging characteristics of a series of retroperitoneal anastomotic hemangiomas, which can be valuable for future diagnoses and help prevent unnecessary surgeries.

## Introduction

Anastomosing hemangioma (AH) is a rare but benign vascular tumor first documented by Montgomery and Epstein in 2009 (1). AH primarily appears in the urogenital tract, particularly in the kidney (2), and exhibits histopathological similarities to highly differentiated angiosarcoma. AH has been observed in various organs, including the renal and adrenal glands, liver, spleen, ovary, testicle, bladder, breast, gastrointestinal tract, retroperitoneum, mesentery/peritoneum, nasal cavity, larynx, left atrium, soft tissue, and bone (3–14). The 2020 WHO classification of Soft Tissue Tumors recognizes AH as a distinct benign vascular neoplasm (15). Retroperitoneal AH is rare, and limited research had been reported, particularly focusing on imaging features. In this study, we present the case of a 53-year-old woman with retroperitoneal AH, which was initially misdiagnosed as ectopic pheochromocytoma, and include an analysis and summary of clinical and imaging features from previously reported cases, which aimed to accurately diagnosing retroperitoneal AH preoperatively and avoiding unnecessary surgical interventions.

## Case presentation

**Figure 1:**
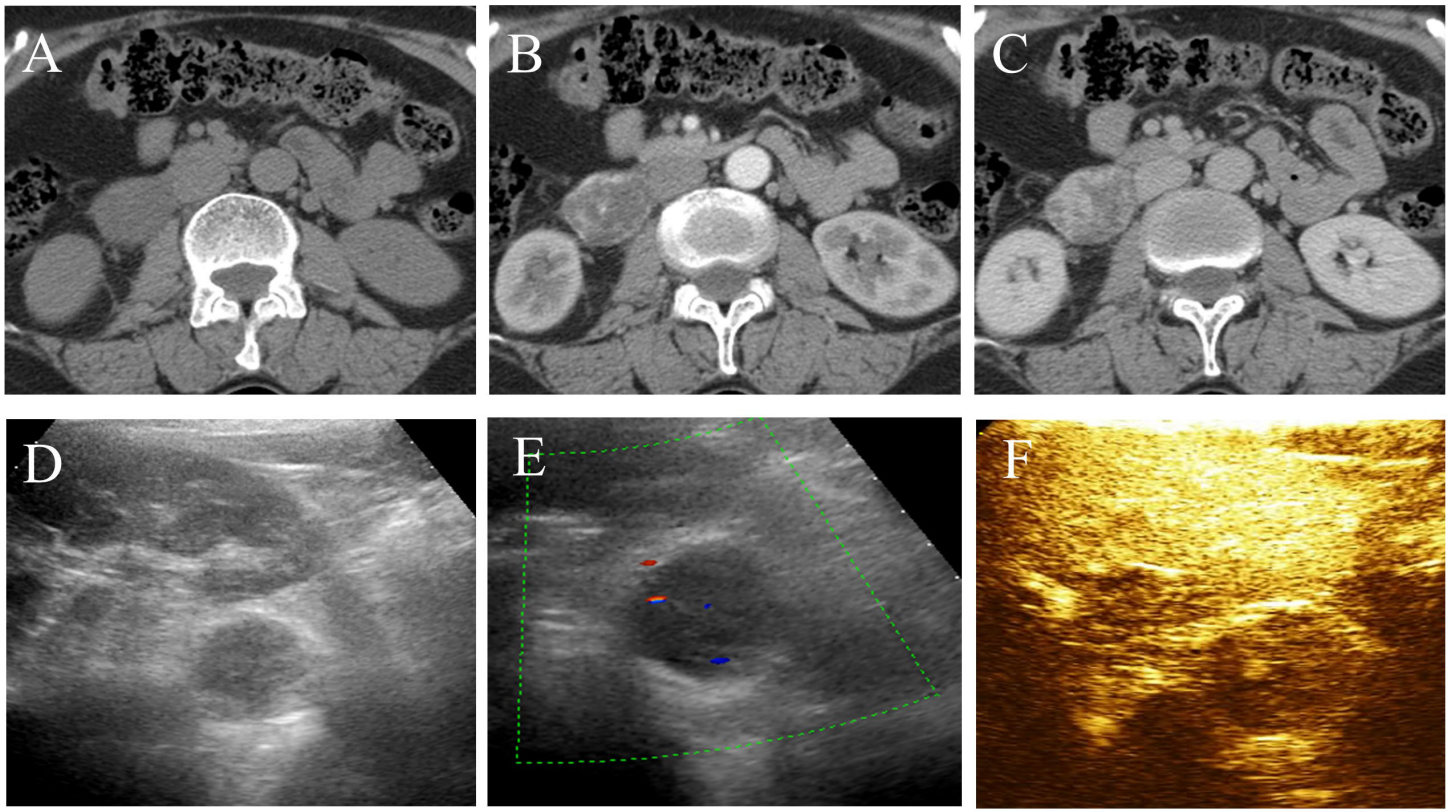
CT and ultrasound features of retroperitoneal anastomosing hemangioma. **(A)** Plain scan. **(B)** Arterial phase. **(C)** Venous phase. **(D, E)** The lesion exhibits a hypoechoic mass with slight blood flow signals in the inferior anteromedial region of the right kidney. **(F)** Contrast-enhanced ultrasound demonstrates homogeneous and “slow-in and slow-out” low enhancement.

**Figure 2:**
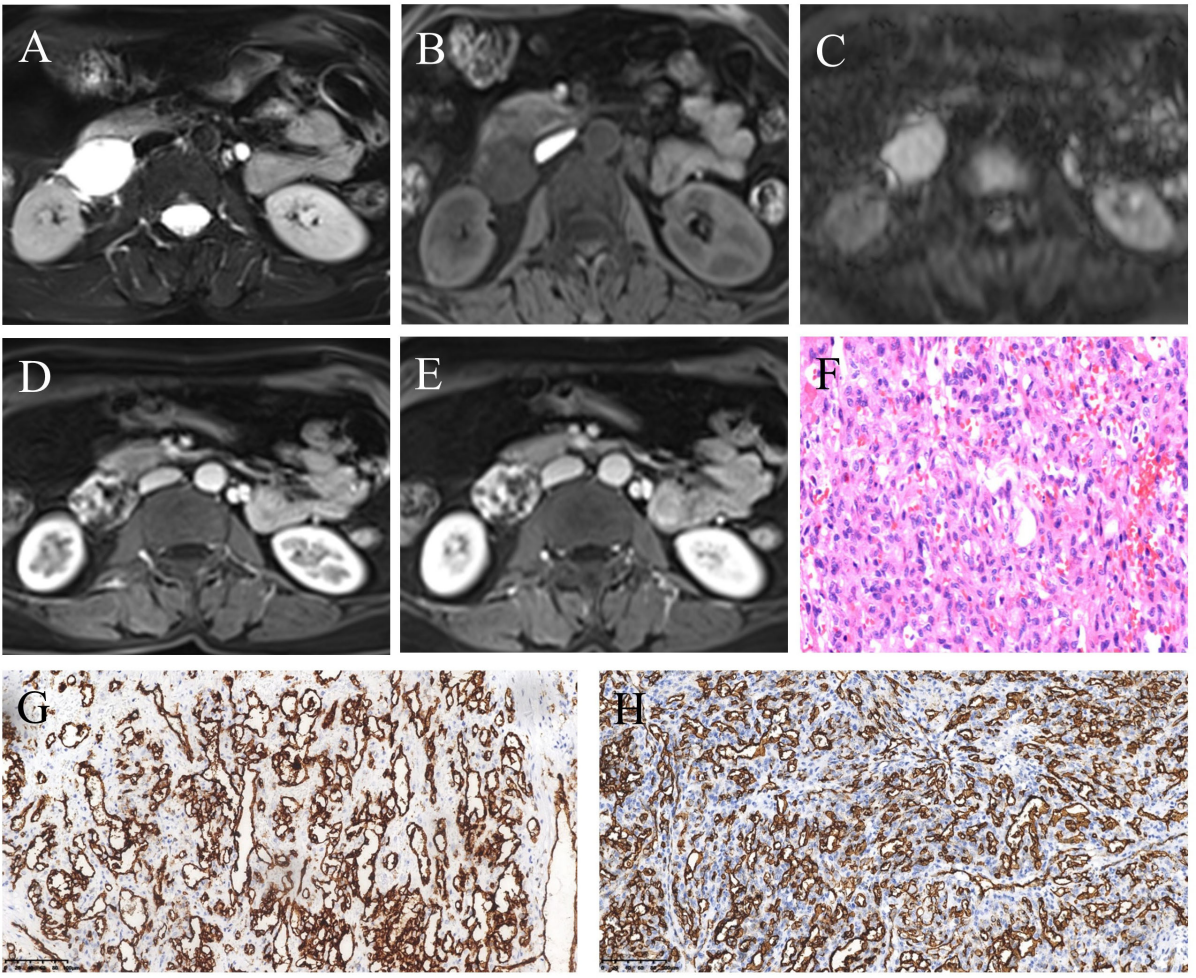
MRI and pathological features of retroperitoneal anastomosing hemangioma. **(A)** T2-weighted image. **(B)** T1-weighted image. **(C)** Diffusion-weighted image. **(D)** Arterial phase post-contrast T1-weighted image. **(E)** Venous phase post-contrast T1-weighted image. **(F)** Hematoxylin and eosin (HE) staining (200×). **(G, H)** Immunohistochemistry (200×) showed vascular endothelial cell markers CD31 **(G)** and CD34 **(H)** were positive.

A 57-year-old woman with left lower back pain for 1 day was admitted to our hospital. The patient had a history of hypertension and hepatitis B infection. Physical examination and laboratory tests, including routine blood and tumor markers, yielded normal results. The outpatient doctor considered that the lower back pain was caused by the kidney. A renal CT scan was performed, revealing a 4.1×3.3×4.8 cm soft tissue density mass in the inferior cavity space of the right kidney. The CT values in the plain scan, arterial phase, and venous phase ranged from approximately 13 to 25 HU, 26 to 174 HU, and 29 to 138 HU, respectively. Notably, the arterial phase demonstrated significant enhancement of the mass, which extended to the center during the venous phase (**Figures 1A–C**). Subsequently, contrast-enhanced ultrasound (CEUS) was performed to further determine whether the lesion is benign or malignant. CEUS revealed a hypoechoic mass in the inferior anteromedial region of the right kidney. The mass displayed a clear boundary and a homogeneous echo signal, and exhibited gradual enhancement during “slow-in and slow-out” phases (**Figures 1D–F**). To further aid diagnosis, a contrast-enhanced magnetic resonance imaging (MRI) scan was conducted. The MRI indicated that the lesion was situated in the right retroperitoneal space, displaying a low signal on T1-weighted imaging (T1WI) and a high signal on T2-weighted imaging (T2WI), with a clear boundary and a slightly increased signal on the diffusion-weighted image (DWI) (**Figures 2A–C**). The enhancement of the mass showed progressive centripetal filling (**Figures 2D, E**). No evidence of adjacent organ invasion or retroperitoneal lymphadenopathy was noted. The preoperative diagnosis was initially considered ectopic pheochromocytoma or angiosarcoma.

During the surgical procedure, the mass was located behind the descending segment of the duodenum, displaying close association with the duodenum and inferior vena cava, along with severe adhesions. Pathological examination revealed a well-defined mass with nodular growth, composed of interconnected and anastomosing blood vessels (**Figure 2F**). Immunohistochemical analysis demonstrated positive staining for CD31 (**Figure 2G**), CD34 (**Figure 2H**), SMA, and focal Ki-67, while Calponin, S-100, Des CK, D2-40, CK5/6, HMB45, Melan-A, NSE, and Melan-A showed negative results. Based on the morphological and immunohistochemical findings, the diagnosis was confirmed as anastomosing hemangioma. The patient has been under follow-up for 40 months, with no recurrence or tumor metastasis observed.

## Discussion

Retroperitoneal AH is an uncommon benign vascular tumor, with only approximately 30 cases reported in the literature (**Table 1**). These cases have been found in the retroperitoneum, para-aortic, paravertebral, and perinephric adipose tissue (1, 4, 10, 12, 16–20). While most of the reported cases focused on pathology or gene mutations, only a few examined the imaging findings.

**Table 1:** Representative reported cases of retroperitoneal AH.

NO.	Authors	Year	Number	Gender/age	Location	Maximum diameter(cm)	CT	MRI	Treatment	Follow-up
1	Montgomery E et al. (1)	2009	1	F/65y	Perinephric adipose tissue	2.0	N/A	N/A	Excision of lesion (without nephrectomy)	8 Months
2	O’Neill AC et al. (16)	2016	7	N/A	Retroperitoneum (NOS)	N/A	N/A	N/A	N/A	N/A
			4*	M/72y	Para-aortic	1.8	Y	N/A	Needle core	N/A
				F/75y	Lateral to the right psoas muscle	5.7	Y	N/A	Needle core	N/A
3	John I et al. (4)	2016	5	M/85y	Para-aortic	4.3	N/A	N/A	Excision	9 Months
				M/69y	Paravertebral	4.2	N/A	N/A	Needle core	N/A
				M/67y	Retroperitoneal	N/A	N/A	N/A	Needle core	2 Months
				M/62y	Paravertebral	2.4	N/A	N/A	Excision	1 Month
				M/53y	Para-aortic	4	Y	N/A	Excision	1 Month
4	Bean GR et al. (17)	2017	1	M/79y	Paravertebral	7.5	N/A	N/A	N/A	8 Months
5	Burton KR et al. (18)	2017	2	M/68y	Right retrocaval	2.0	Y	N/A	Excision	24 Months
					Left perirenal fat	1.5	Y	N/A	Excision	24 Months
6	Jayaram A et al. (19)	2018	1	F/53y	Left paravertebral region	4.5	N/A	N/A	Excision	N/A
7	Liau JY et al. (12)	2020	7	F/70y	Retroperitoneum	6.5	N/A	N/A	N/A	N/A
				M/63y		2.7	N/A	N/A	N/A	N/A
				F/69y		2.7	N/A	N/A	N/A	N/A
				M/33y	Para-aortic	8	N/A	N/A	N/A	N/A
				M/79y	Para-aortic	1.3	N/A	N/A	N/A	N/A
				F/77y	Para-spinal	4	N/A	N/A	N/A	N/A
				F/61y	Para-spinal	4.8	N/A	N/A	N/A	N/A
8	Zheng LP et al. (20)	2020	1	F/74y	Left renal vein	2.6	Y	Y	Laparoscopic resection	2 Months
9	Xue X et al. (10)	2022	1	F/64y	Retroperitoneum	10.8	Y	Y	Laparoscopic resection	24 Months

^*^No details were available for the remaining two cases. N/A, information not available; AH, anastomosing hemangioma.

Previous literature indicates that AH predominantly affects men and typically presents asymptomatically, with a few cases showing low back pain, intermittent hematuria, and lower urinary tract symptoms. However, retroperitoneal AH often lacks specific clinical symptoms and signs, and it is frequently discovered incidentally during physical examinations (5). This is likely due to its deep retroperitoneal location, which hinders the appearance of symptoms. Among the 30 retroperitoneal cases collected in **Table 1**, there is a male-to-female ratio of approximately 1.2:1, with a slightly higher occurrence in men. The case that we report involves a middle-aged woman who presented with left low back pain, even though the lesion was located in the right retroperitoneal space. With a detailed history, we think that the lower back pain may be associated with muscle injury after physical labor, not the lesion. The age of AH patients ranges from 1 to 85 years old (14), with the median age for extrarenal AH being 65 years old. For retroperitoneal AH, the summarized age range is from 33 to 85 years old (average 66.9 ± 11.5 years) (**Table 1**), and the age in this case was 57 years old. Therefore, retroperitoneal AH appears to be more prevalent among the elderly. Existing literature reports a wide range of AH sizes, varying from 0.1 cm to 14 cm (14). According to the limited available data, the size of retroperitoneal AH ranges from 1.3 to 10.8 cm (average 4.2 cm). The maximum diameter in our case was 4.8 cm, consistent with previous reports. The imaging findings of reported AH cases have been limited, particularly in the retroperitoneal space, where only six patients with seven cases have been reported (**Table 2**). The ultrasound features of AH have not been extensively described in the literature. AH appears as lesions with a clear boundary and mixed echogenicity, and color Doppler flow imaging (CDFI) may show intense internal vascularity (5). The ultrasound characteristics of retroperitoneal AH were previously reported only by Xue et al. (10), who described it as a hypoechoic mass with abundant blood flow signals in CDFI. In our case, we observed a hypoechoic mass with some heterogeneity and a slight presence of blood flow, which differed from the previous study. Contrast-enhanced ultrasound revealed “slow-in and slow-out” with slight heterogeneous enhancement. Most AH cases typically appeared as low or equal density on CT scans, with a plain scan CT value of approximately 27–35 HU (11, 21). In contrast, the reported plain scan CT value for retroperitoneal AH ranged from approximately 15 HU to 37 HU, with our case measuring approximately 13–25 HU. Enhanced CT scans showed evident heterogeneous enhancement in the arterial phase for most lesions, followed by progressive enhancement in the venous phase, reaching values of 157–265 HU, similar to the aorta. In our case, the highest enhanced CT value was 174 HU, with visible nourishing vessels. Although the degree of enhancement decreased in the venous phase, it displayed centripetal filling enhancement. Variations in CT values may be associated with the number of vascular components within the lesion. Some AHs may exhibit unenhanced components, possibly due to surrounding fat wrapping or unenhanced areas resulting from degeneration or intravascular thrombosis. Xue et al. (10) reported the presence of a capsule in their case, but no other retroperitoneal AHs have been reported with this feature, suggesting the need for further verification. On MRI, typical AH appeared as a low signal on T1WI and a significantly high signal on T2WI. Additionally, there was evident enhancement in the arterial phase, followed by persistent centripetal filling in the venous phase. This enhancement pattern resembled that of cavernous hemangioma. DWI showed isointensity to high intensity in other locations (3, 20). In our study, DWI exhibited slightly high intensity, possibly due to the small number of cells and their loose arrangement. Merritt et al. (3) described the MRI imaging findings of intrahepatic AH, which showed progressive enhancement. This suggests that the enhancement features of AH in different locations may be similar. This case report contained multimodal imaging (ultrasound, CT, and MRI); enhancements of these examinations suggested that the mass may be a vascular neoplasm.

**Table 2:** Imaging features of seven* pathologically proven cases of retroperitoneal AH.

Features	O’Neill AC et al.		John I et al.	Burton KR et al.		Zheng LP et al.	Xue X et al.
Location	Para-aortic(nodule)	Lateral to the right psoas muscle	Para-aortic	Right retrocaval	Left perirenal fat	Left renal vein	Retroperitoneum
Ultrasound appearance	N/A	N/A	N/A	N/A	N/A	N/A	Hypoechoic mass, abundant blood flow signals
Contour	Well-defined	Not well-defined	Not well-defined	Not well-defined	Well-defined	Not well-defined	Well-defined
Pre-contrast	Hyperdense	Hypodense	N/A	N/A	N/A	Hypodense	Hypodense
CT value							
NC	32HU	15HU	N/A	N/A	N/A	N/A	17–36HU
PC	157HU	N/A	N/A	N/A	N/A	N/A	111–173HU(AP) 232–265HU(VP)
Heterogeneity	Homogeneous	Heterogeneous	Heterogeneous	Heterogeneous	Heterogeneous	Heterogeneous	Heterogeneous
CT Enhancement	N/A	N/A	N/A	N/A	N/A	Persistent enhancement	Progressive enhancement
MRI							
T1WI	N/A	N/A	N/A	N/A	N/A	Hypointensity	Hypointensity
T2WI	N/A	N/A	N/A	N/A	N/A	Hyperintensity	Iso-hyper intensity
DWI	N/A	N/A	N/A	N/A	N/A	Hyperintensity	Isointensity
MRI Enhancement	N/A	N/A	N/A	N/A	N/A	Persistent enhancement	Obvious progressive enhancement

^*^No imaging data were available for the remaining six cases. AH, anastomosing hemangioma; AP, arterial phase; DWI, diffusion weighted imaging; HU, Hounsfield units; N/A, information not available; NC, non-contrast; PC, post-contrast; T1WI, T1-weighted imaging; T2WI, T2-weighted imaging; VP, venous phase.

Retroperitoneal AH needs to be differentiated from the following diseases. First is ectopic pheochromocytoma, where most patients have rapid changes in blood pressure and a history of hypertension; it is prone to necrosis and cystic degeneration, with an obvious enhancement and slow washout. Second, retroperitoneal angiosarcoma is generally large and infiltrates the surrounding structures; it demonstrates a relatively high degree of malignancy and displays rapid short-term growth.

AH generally carries a favorable prognosis and is considered a benign lesion. No reports of metastasis or recurrence have been documented after resection. The longest follow-up for retroperitoneal AH has been 24 months without recurrence. In our case, the follow-up period was 40 months with no recurrence observed. However, it is important to note that the report of Burton et al. (18) indicated that multifocality or recurrence may still be possible.

## Conclusion

Accurately diagnosing retroperitoneal AH preoperatively can be challenging due to its low incidence. Based on a summary of available imaging cases, AH should be considered in elderly patients exhibiting strong arterial phase enhancement with subsequent centripetal filling. AH is typically benign and slow growing, and early identification of this tumor may allow for appropriate follow-up, potentially avoiding unnecessary surgical interventions.

## Data availability statement

The raw data supporting the conclusions of this article will be made available by the authors, without undue reservation.

## Ethics statement

Written informed consent was obtained from the individual(s) for the publication of any potentially identifiable images or data included in this article.

## Author contributions

LZ: Conceptualization, Funding acquisition, Writing – original draft, Writing – review & editing. JW: Data curation, Resources, Writing – original draft.
